# Supplementary material for: Fitness benefits in fluoroquinolone-resistant Salmonella Typhi in the absence of antimicrobial pressure
Source: eLife. 2013 Dec 10;2:e01229. doi: 10.7554/eLife.01229 (PMC3857714; doi:10.7554/eLife.01229)
Supplement: Supplementary file 1. [file elife01229s001.docx]

**Supplementary File 1. Oligonucleotides used in this study**

| **Primer name** | **Sequence (5’→3’)** | **Primer function/target** |
| --- | --- | --- |
| 47125 | TTCATTATGGTGAAAGTTGGAACC | Screening of BRD948 *gyrA*::pWT-*gyrA* derivatives and  BRD948 *aroC*::pWT-*aroC* derivatives * |
| Cat-12 | CCATAATCAGTCGACGGTATCGATAAGCTT | Amplification of pJCB12 fragment |
| GyrA-07 | GGGTCGACTGATTATGGTTTATGCCTCC | Construction of all *gyrA* mutations * |
| GyrA-10 | GCGCATGCCACGACCGGTACGGTAG | Construction of all *gyrA* mutations * |
| GyrA-11 | TCGCGTCAGCTTCAACTTCC | Screening of BRD948 *gyrA*::pWT-*gyrA* derivatives * |
| GyrA-8 | TCATACACTGCG**T**AATCGCCGTGGGGATG | Construction of *gyrA* S83Y * |
| GyrA-9 | CCCACGGCGATT**A**CGCAGTGTATGACACC | Construction of *gyrA* S83Y * |
| GyrA-32 | TCATACACTGCG**A**AATCGCCGTGGGGATG | Construction of *gyrA* S83F * |
| GyrA-33 | CCCACGGCGATT**T**CGCAGTGTATGACACC | Construction of *gyrA* S83F * |
| GyrA-41 | AACGATGGTGT**T**ATACACTGCGGAATC | Construction of *gyrA* D87N * |
| GyrA-42 | TTCCGCAGTGTAT**A**ACACCATCGTTCG | Construction of *gyrA* D87N * |
| GyrA-34 | AACGATGGTGT**T**ATACACTGCG**A**AATC | Construction of *gyrA* S83F and D87N * |
| GyrA-35 | TT**T**CGCAGTGTAT**A**ACACCATCGTTCG | Construction of *gyrA* S83F and D87N * |
| GyrA-47 | AACGATGGTGC**C**ATACACTGCG**A**AATC | Construction of *gyrA* S83F and D87G |
| GyrA-48 | TT**T**CGCAGTGTAT**G**GCACCATCGTTCG | Construction of *gyrA* S83F and D87G |
| GyrA-49 | CGATGGTG**G**CATACACTGCGAAATC | Construction of *gyrA* D87A |
| GyrA-50 | CGCAGTGTATG**C**CACCATCGTTCG | Construction of *gyrA* D87A |
| GyrA-51 | CGATGGTG**C**CATACACTGCGAAATC | Construction of *gyrA* D87G |
| GyrA-52 | CGCAGTGTATG**G**CACCATCGTTCG | Construction of *gyrA* D87G |
| GyrA-53 | AACGATGGTG**G**CATACACTGCG**A**AATC | Construction of *gyrA* S83F and D87A |
| GyrA-54 | TT**T**CGCAGTGTATG**C**CACCATCGTTCG | Construction of *gyrA* S83F and D87A |
| ParC-18 | GCGGTCGACGTTGGCGTGGTGAATAG | Construction of all *parC* mutations |
| ParC-21 | CGCGCATGCGGCAACGCGGTGATCA | Construction of all *parC* mutations |
| ParC-23 | GCCAGCAGTCAAACTTTTGG | Screening of BRD948 *parC*::pWT-*parC* derivatives |
| ParC-27 | ATAGCAGGCGATGTCGCCATG | Construction of *parC* S80I mutation |
| ParC-28 | CATGGCGACATCGCCTGCTAT | Construction of *parC* S80I mutation |
| R6K-01 | GTGACACAGGAACACTTAACGGC | Screening of BRD948*-parC::*pWT*-parC* derivatives |
| R6K-03P | P-CCATGTCAGCCGTTAAGTGTTC | Amplification pJCB12 fragment |
| aroC-1 | ATGTCGACCCACTTCGCTGGCCTTATTAG | Construction of all *aroC* mutation |
| aroC-2 | GTGCATGCGGCAAAGCTACCGATAGACT | Construction of all *aroC* mutation |
| aroC-3 | GAGTTGTCCAATTG**A**GTTTCCTGCCATC | Construction of *aroC* mutation **A**CA→ **T**CA |
| aroC-4 | GGCAGGAAAC**T**CAATTGGACAACTCTTTCG | Construction of *aroC* mutation **A**CA → **T**CA |
| aroC-5 | CGAAATAACGGCGCTGATCGGTG | Screening for BRD 948 *aroC*::pWT-*aroC* derivatives |
| pyro-gyrA83-F | TGGGCAATGACTGGAACAAAG | Pre-pyrosequencing amplification of *gyrA* codon 83 |
| pyro-gyrA83-R | biotin-TACCGTCATAGTTATCCACG | Pre-pyrosequencing amplification of *gyrA* codon 83 |
| pyro-gyrA83-S | CGGTAAATACCATCCCCA | Pyrosequencing of *gyrA* codon 83 |
| pyro-gyrA87-F | TGGGCAATGACTGGAACAAAG | Pre-pyrosequencing amplification of *gyrA* codon 87 |
| pyro-gyrA87-R | biotin-TACCGTCATAGTTATCCACG | Pre-pyrosequencing amplification of *gyrA* codon 87 |
| pyro-gyrA87-S | CGATTCCGCAGTGTA | Pyrosequencing of *gyrA* codon 87 |
| pyro-parC80-F | biotin-ACCGTTGGCGACGTACTGG | Pre-pyrosequencing amplification of *parC* codon 80 |
| pyro-parC80-R | AATTCCCCTGGCCATCGAC | Pre-pyrosequencing amplification of *parC* codon 80 |
| pyro-parC80-S | CATGGCTTCATAGCAG | Pyrosequencing of *parC* codon 80 |
| pyro-aroC-F | Biotin-GGCCCGTGAACATTTCAA | Pre-pyrosequencing amplification of *aroC* |
| pyro-aroC-R | GTTACGCGAAAGAGTTGTCCA | Pre-pyrosequencing amplification of *aroC* |
| pyro-aroC-S | CGAAAGAGTTGTCCAAT | Pyrosequencing of *aroC* |

***** As described in Turner *et al.* Nucleotides in which a substitution was induced are in bold and SalI and SphI restriction endonuclease sites used in pJCB12 ligation are underlined
